# Diamagnetic Signature of Beta‐Amyloid (Aβ) and Tau (τ) Tangle Pathology in Alzheimer's Disease: A Review

**DOI:** 10.1002/agm2.70006

**Published:** 2025-02-09

**Authors:** Sadegh Ghaderi, Sana Mohammadi, Farzad Fatehi

**Affiliations:** ^1^ Neuromuscular Research Center, Department of Neurology, Shariati Hospital Tehran University of Medical Sciences Tehran Iran; ^2^ Department of Neuroscience and Addiction Studies, School of Advanced Technologies in Medicine Tehran University of Medical Sciences Tehran Iran; ^3^ Neurology Department University Hospitals of Leicester NHS Trust Leicester UK

**Keywords:** Alzheimer's disease, diamagnetic properties, quantitative susceptibility mapping

## Abstract

The complex interplay between diamagnetic and paramagnetic substances within the brain, particularly in the context of Alzheimer's disease (AD), offers a rich landscape for investigation using advanced quantitative neuroimaging techniques. Although conventional approaches have focused on the paramagnetic properties of iron, emerging and promising research has highlighted the significance of diamagnetic signatures associated with beta‐amyloid (Aβ) plaques and Tau (τ) protein aggregates. Quantitative susceptibility mapping (QSM) is a complex post‐processing technique that visualizes and characterizes these subtle alterations in brain border tissue composition, such as the gray–white matter interface. Through voxel‐wise separation of the contributions of diamagnetic and paramagnetic sources, QSM enabled the identification and quantification of Aβ and τ aggregates, even in the presence of iron. However, several challenges remain in utilizing diamagnetic signatures of Aβ and τ for clinical applications. These include the relatively small magnitude of the diamagnetic signal compared to paramagnetic iron, the need for high‐resolution imaging and sophisticated analysis techniques, and the standardization of QSM acquisition and analysis protocols. Further research is necessary to refine QSM techniques, optimize acquisition parameters, and develop robust analysis pipelines to improve the sensitivity and specificity of detecting the diamagnetic nature of Aβ and τ aggregates. As our understanding of the diamagnetic properties of Aβ and τ continues to evolve, QSM is expected to play a pivotal role in advancing our knowledge of AD and other neurodegenerative diseases.

## Graphical abstract

Alzheimer's disease (AD) is characterized by the accumulation of amyloid‐beta (Aβ) plaques and tau (τ) tangles in the brain. While iron accumulation has been proposed and studied in AD, recent research has highlighted the significance of diamagnetic signatures associated with Aβ and τ aggregates. Quantitative susceptibility mapping (QSM) is a powerful technique that can detect these diamagnetic changes. By separating paramagnetic and diamagnetic components of the susceptibility signal, QSM allows for the quantification of Aβ and τ burden in the brain.

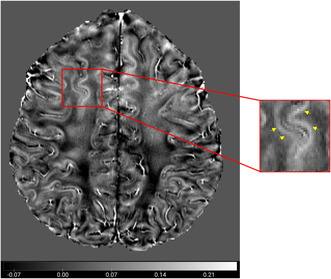

## 1 Introduction

Neurological disorders and aging are associated with myriad alterations in brain composition. These alterations can involve the accumulation of minerals, such as iron, or proteins, such as beta‐amyloid (Aβ) plaques and Tau protein (τ) tangles of [1]. Additionally, modifications in the lipid and myelin content of the brain can significantly affect neuronal function. Age‐related myelin breakdown and demyelination can impair nerve conduction and contribute to cognitive decline [2].

Alzheimer's disease (AD) is a prevalent major issue in global health and age‐related dementia and is characterized by progressive cognitive decline [3, 4]. The pathological features of the disease include the accumulation of two abnormal protein structures in the brain: Aβ plaques and τ tangles [5]. While the role of Aβ in AD development is debated, both Aβ and τ proteinopathies are thought to trigger a cascade of detrimental events, leading to neuronal damage and neurodegeneration [6]. These events include oxidative stress, microvascular dysfunction, blood–brain barrier disruption, and inflammatory responses [4].

Aβ plaques are formed by the aggregation of Aβ peptides and fragments derived from amyloid precursor proteins (APPs). Although the precise role of Aβ in AD development remains debated, growing evidence suggests that Aβ aggregation triggers a series of detrimental events, including oxidative stress, inflammation, and disruption of neuronal function, ultimately leading to cell death [7]. τ neurofibrillary tangles (NFTs) result from the hyperphosphorylation and aggregation of τ protein, a microtubule‐associated protein that is crucial for maintaining neuronal structure and function. The abnormal accumulation of τ disrupts cellular transport mechanisms, impairs synaptic communication, and contributes to neuronal dysfunction and loss [8]. Adding complexity to AD pathology is the abnormal accumulation of iron in the brain, a phenomenon observed in the early stages of the disease that is strongly associated with both Aβ plaques and τ tangles [9]. Iron, which is essential for various brain functions, is detrimental when it accumulates in excess. Excess iron can promote Aβ and τ aggregation, and iron can directly bind to Aβ and τ, facilitating their aggregation and enhancing their toxicity. Exacerbate oxidative stress: Iron, particularly in its ferrous (Fe^2+^) form, participates in the Fenton reaction, generating harmful reactive oxygen species (ROS) that damage cellular components and contribute to neuronal death [10]. Trigger ferroptosis, an iron‐dependent form of cell death characterized by lipid peroxidation, is increasingly being recognized as a contributor to AD neurodegeneration [11]. Thus, the interplay between iron deposition and protein aggregation underscores the complex and multifaceted nature of the AD pathology. While Aβ and τ are central to the disease process, iron dysregulation significantly contributes to their aggregation, toxicity, and subsequent neuronal damage, ultimately driving AD progression.

The human brain is a complex organ with a heterogeneous composition of substances, each possessing unique magnetic properties that can be broadly categorized as either diamagnetic or paramagnetic [12]. Accumulation of iron, a *paramagnetic* element, is observed in the early stages of AD and is associated with both Aβ and τ (especially abnormally phosphorylated τ (p‐τ)) [5, 13]. Diamagnetic substances, such as water, myelin, and proteins such as Aβ and τ, weakly repel magnetic fields. Conversely, paramagnetic substances, such as iron, are attracted to magnetic fields [14, 15]. These inherent magnetic properties offer valuable insights into brain tissue composition and can be harnessed using advanced neuroimaging techniques to non‐invasively probe microstructural alterations associated with AD [16, 17].

Recent studies have highlighted the importance of *diamagnetic* signatures, particularly those associated with Aβ and τ aggregates [9, 18]. Previous clinical [19, 20], phantom [21], postmortem [16], and animal [22] studies have shown that Aβ aggregates are diamagnetic and can be visualized using quantitative susceptibility mapping (QSM). QSM allows the measurement of magnetic susceptibility (χ), which can be used to differentiate diamagnetic and paramagnetic substances [23, 24]. The diamagnetic nature of Aβ and τ contributes to the contrast observed in QSM images, providing valuable insights into the distribution and progression of amyloid pathology [19]. Although Aβ and τ are diamagnetic, their aggregation and normal aging may lead to accelerated demyelination, which can increase paramagnetism [25]. In addition to studying iron deposition, QSM has been applied to investigate demyelination and the length of myelinated fibers in white matter tracts, which are characterized by a decrease in diamagnetic myelin and result in increased (less negative) susceptibility values. The ability of QSM to detect both paramagnetic and diamagnetic changes makes it a versatile tool for studying a wide range of neurological disorders [19, 21, 25, 26].

Investigating the diamagnetic properties of Aβ plaques and τ tangles may offer new insights into the pathophysiological nature of AD. Thus, we aimed to discuss the diamagnetic signatures of Aβ and τ in AD and highlight their potential as diagnostic and management targets.

### 1.1 Pathophysiology of AD

#### 1.1.1 Role of Beta‐Amyloid (Aβ) and Tau (τ) Tangle

Aβ is a peptide derived from the APP through sequential cleavage by β‐secretase (BACE1) and γ‐secretase. Aβ peptides, particularly longer Aβ42, are prone to aggregation, leading to the formation of oligomers, protofibrils, fibrils, and ultimately mature amyloid plaques. Aβ accumulation can begin decades before the onset of clinical symptoms [27, 28]. These aggregates disrupt neuronal function through various mechanisms, including synaptic dysfunction, mitochondrial dysfunction, oxidative stress, inflammation, and cerebral amyloid angiopathy (CAA) [29].

τ is a microtubule‐associated protein that plays a critical role in stabilizing the microtubules, which are essential for neuronal structure and function. In AD, τ undergoes hyperphosphorylation, detachment from microtubules, and NFT formation. These tangles disrupt axonal transport, impair neuronal communication, and cause cell death. The spread of τ pathology is thought to follow a specific pattern, progressing from the transentorhinal region to the hippocampus and neocortex. Τau tangles can disrupt synapses, impair axonal transport, and contribute to mitochondrial dysfunction. Additionally, τ aggregates can spread from neuron to neuron, potentially contributing to the progression of pathology throughout the brain [30, 31].

Although the amyloid cascade hypothesis initially posits that Aβ accumulation triggers the subsequent formation of τ tangles, recent evidence suggests a more complex interplay between these two pathologies [9]. Studies have found that τ pathology may be a better correlate of cognitive decline than Aβ burden [19, 22]. Additionally, iron accumulation, a prominent feature of AD, can interact with both Aβ and τ, potentially exacerbating their aggregation and toxicity [32]. Aβ may induce τ hyperphosphorylation and aggregation through the activation of kinases such as CDK5. Conversely, τ pathology may enhance Aβ toxicity by impairing its clearance or contributing to synaptic dysfunction. Iron accumulation can promote both Aβ and τ aggregation, potentially serving as a crucial link between these two pathologies [33].

#### 1.1.2 Role of Iron

Iron plays a multifaceted role in the pathophysiology of AD [34]. Although it is essential for various brain functions, iron accumulation is a hallmark feature of AD and is linked to disease progression and severity [33]. This accumulation is particularly evident in brain regions vulnerable to AD pathology, such as the hippocampus, frontal cortex, and temporal lobes [25, 33]. Iron is found within Aβ and τ protein aggregates, potentially facilitating their formation and enhancing their toxicity.

Moreover, iron is a key player in inflammation, a crucial process in AD. Activated microglia, the immune cells of the brain, accumulate iron and contribute to neuroinflammation and neuronal damage. Iron, particularly in its ferrous (Fe^2+^) form, is highly reactive and generates harmful free radicals through the Fenton reaction. These ROS induce oxidative stress, which is a major contributor to neuronal damage and cell death in AD. Iron overload can accelerate AD progression, as evidenced by the increased risk of dementia in individuals with hereditary hemochromatosis. Iron accumulation may also contribute to cell death via ferroptosis, an iron‐dependent form of cell death characterized by lipid peroxidation [1, 9, 16, 20, 33].

Iron dysregulation in AD involves altered levels of iron‐related proteins, including transferrin, ferritin, ferroportin, and hepcidin. These proteins regulate iron uptake, storage, and export in the brain, and their dysregulation contributes to iron accumulation and the pathological changes seen in AD [33, 35, 36].

The specific mechanisms through which iron contributes to AD pathology are complex and multifaceted. Iron‐catalyzed oxidative stress damages cellular components, including proteins, lipids, and DNA, leading to cellular dysfunction and death. Ferroptosis, an iron‐dependent form of cell death, is characterized by lipid peroxidation and is implicated in AD neurodegeneration. Iron can bind to Aβ peptides and promote their aggregation into toxic plaques. It can also interact with the τ protein, facilitating its hyperphosphorylation and the formation of NFTs. Iron accumulation in activated microglia exacerbates inflammation, which contributes to neuronal damage. Additionally, iron overload can disrupt vital cellular processes, including mitochondrial function, energy production, and neurotransmitter synthesis. Iron may also contribute to blood–brain barrier dysfunction, leading to increased permeability and neurotoxicity [37, 38, 39].

Thus, iron accumulation appears to be a central player in the complex pathophysiological cascade of AD, interacting with both Aβ and τ pathologies, and contributing to oxidative stress and neuroinflammation.

### 1.2 Diamagnetic Signature of Aβ and Tau

The co‐localization of iron with Aβ plaques, often visualized as hypointensities on T2‐weighted images, has led to the development of techniques such as QSM to quantify and map iron deposition in AD [18]. QSM studies have shown that increased iron deposition in specific brain regions, particularly the hippocampus and basal ganglia, correlates with cognitive decline in AD patients [9, 21, 33, 34]. However, the presence of iron can also complicate the detection of diamagnetic Aβ and τ signatures, requiring advanced source separation methods to isolate their contributions [1, 40].

QSM produces a map of magnetic susceptibilities and provides valuable insights into brain tissue composition. Negative values on a QSM map represent diamagnetic properties, while positive values indicate paramagnetic properties relative to a chosen tissue reference [1, 41, 42]. Unlike T2*‐weighted imaging, QSM is quantitative, reflects local tissue properties, is more sensitive to susceptibility effects, and is less dependent on imaging parameters and object orientation. Negative values on the QSM map indicate diamagnetic properties, whereas positive values suggest paramagnetic properties relative to a tissue reference. This technique has been used to study susceptibility changes associated with iron, calcium, myelin, or lipids in various neurological diseases. Although QSM is susceptible to artifacts, it offers a valuable tool for the precise assessment of brain tissue composition and the detection of subtle changes in susceptibility [43].

The pathophysiology of AD is intricately linked to the accumulation of Aβ plaques, *τ* tangles, and iron in the brain. QSM studies of AD have focused on iron accumulation, a paramagnetic substance that strongly attracts magnetic fields. However, recent research has revealed that Aβ and *τ*, which are primarily diamagnetic, may also contribute to detectable changes in MRI signals. QSM can detect subtle changes in susceptibility caused by diamagnetic materials, making it a potential tool for visualizing Aβ and τ aggregates. Studies have demonstrated that Aβ plaques are diamagnetic and produce strong contrast on susceptibility maps obtained through QSM [20, 21, 41]. Longitudinal studies using QSM have shown the potential of monitoring Aβ accumulation in mouse models of AD [44]. Post‐mortem brain specimens from AD patients with AD further validated the diamagnetic properties of Aβ [18, 45].

On the other hand, one significant challenge in utilizing the diamagnetic signature of Aβ and τ is the presence of iron, a paramagnetic substance that often co‐localizes with Aβ plaques [20, 34, 46]. Iron can counteract the diamagnetic signal of Aβ, reducing its visibility on QSM maps. To address this challenge, advanced QSM techniques have been developed to separate diamagnetic and paramagnetic components of susceptibility signals. This source separation allows researchers to isolate the contribution of diamagnetic species, such as Aβ and τ, from the influence of iron, thereby enhancing the sensitivity and specificity of detecting these protein aggregates (see *QSM Advances*).

### 1.3 QSM Advances

QSM is a noninvasive MRI probe technique that enables the quantification of χ in the brain. This technique is particularly valuable for separating and quantifying the contributions of diamagnetic and paramagnetic sources, which can be challenging to distinguish using conventional MRI methods [26, 47]. AD is characterized by the accumulation of Aβ plaques and τ tangles, both of which are diamagnetic. However, the presence of iron, a paramagnetic substance that often co‐localizes with Aβ plaques, can complicate the detection of these diamagnetic proteins using MRI [9]. QSM's ability to separate these opposing magnetic signals is crucial for accurately quantifying the contribution of each source and gaining a clearer understanding of the underlying pathology using complex post‐processing steps such as masking, phase unwrapping, background field removal, and map reconstruction of a multiecho gradient‐recalled echo (GRE) [47, 48, 49].

One of the primary hurdles lies in the coexistence of multiple susceptibility sources within a single voxel [50]. While Aβ and τ exhibit diamagnetic properties, their signals can be obscured by the stronger paramagnetic effects of iron, which is often found in close proximity to Aβ plaques. Several QSM techniques have been developed to disentangle the contributions of paramagnetic and diamagnetic sources, including χ‐separation [12], DECOMPOSE‐QSM [51], APART‐QSM [52], and QSM with adaptive relaxometric constant estimation (QSM‐ARCS) [53]. These techniques leverage multi‐echo gradient echo (GRE) data to model signal evolution and separate the paramagnetic (e.g., iron) and diamagnetic (e.g., calcium, myelin, and lipids) components of the susceptibility signal. This holds promise for improving the understanding of the complex interplay between iron, myelin, and pathological protein aggregates in AD and other neurodegenerative diseases [54].

In summary, the χ‐separation (or chi‐separation) technique [12] leverages a biophysical model that considers the linear dependence of the reversible transverse relaxation rate (R2') on absolute magnetic susceptibility. By combining magnitude and phase information from multi‐echo GRE data, χ‐separation generates separate maps of paramagnetic and diamagnetic susceptibility. The DECOMPOSE‐QSM method [51] models signal evolution using three‐pool complex exponentials based on multi‐echo GRE data. It calculates separate maps of paramagnetic component susceptibility (PCS) and diamagnetic component susceptibility (DCS). The APART‐QSM [52] iterative data fitting method improved the sub‐voxel‐level quantification of susceptibility sources, facilitating their separation. A recent novel QSM‐ARCS method [53] separates paramagnetic and diamagnetic sources from the GRE. This method accurately estimated the opposing susceptibilities, as validated against a method using R2 and R2* maps. Significant correlations between these susceptibilities and fractional anisotropy suggest their potential as biomarkers for myelin and iron content. Additionally, the authors observed fiber orientation dependencies, indicating that these susceptibilities can provide insights into the underlying magnetic sources and their biological implications.

Therefore, the diamagnetic susceptibility map derived from QSM source separation has been shown to be a promising biomarker. Studies have demonstrated a strong correlation among diamagnetic susceptibility, Aβ plaques, and τ angles. This suggests that sub‐voxel diamagnetic susceptibility can be used to assess AD and detect subtle alterations.

However, it is important to note that a diamagnetic susceptibility map is an indirect measure of diamagnetic content and should not be directly equated to a density map. The accuracy of QSM source separation methods can be affected by factors, such as high susceptibility concentrations and differences in susceptibility characteristics between different sources. Additionally, standardization of the QSM acquisition and analysis protocols is essential to ensure consistent and reliable results [55].

## 2 Discussion

**FIGURE 1:**
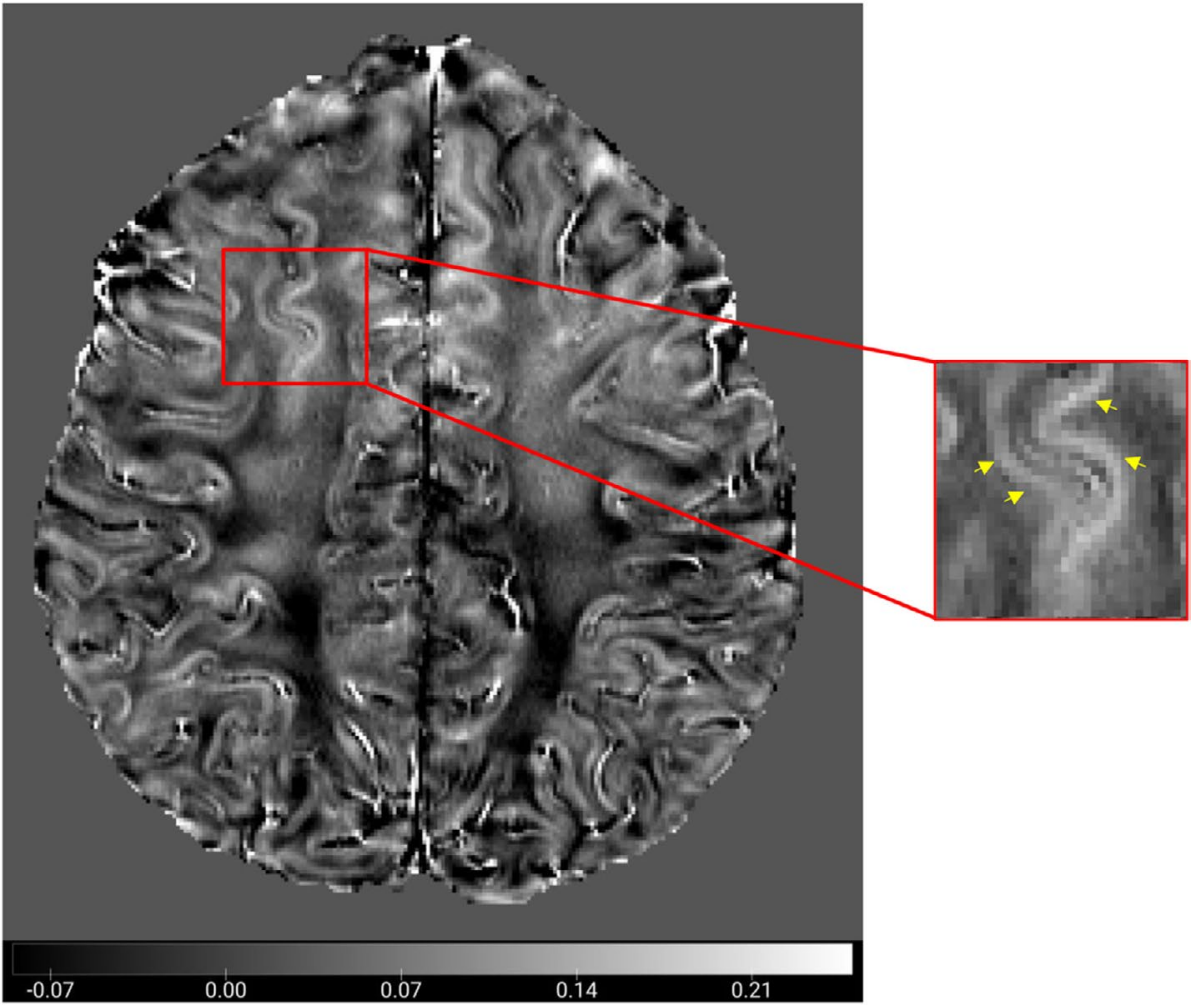
Quantitative susceptibility mapping (QSM) demonstrated the interface between the gray and white matter in healthy subjects. The yellow arrow highlights the region of interest where both paramagnetic and diamagnetic properties can be observed.

Positron emission tomography (PET) imaging is the gold standard for detecting Aβ and τ proteins in the brain using tracers such as the Pittsburgh compound (PiB) and Flortaucipir [56, 57], respectively, and is limited by factors such as low spatial resolution, radiation exposure, and off‐target binding [58]. The current emerging literature suggests that QSM holds promise in elucidating the pathophysiological mechanisms underlying AD by revealing sub‐voxel alterations in susceptibility between diamagnetic and paramagnetic interfaces. Considering the relative concentrations of paramagnetic and diamagnetic substances in gray matter, an increase in susceptibility is predominantly attributed to elevated iron levels. In contrast, in white matter, where diamagnetic myelin predominates, an increase in magnetic susceptibility may arise from a combination of demyelination and iron deposition [48] (Figure 1).

White matter integrity is crucial for efficient communication in the brain. In AD, white matter undergoes significant demyelination and atrophy, which contributes to cognitive impairment. Myelin, the fatty sheath surrounding nerve fibers, is a major diamagnetic component of white matter [19, 44, 59]. Therefore, demyelination leads to a reduction in the diamagnetism. This reduction in diamagnetic susceptibility can serve as a marker for white matter damage in AD.

QSM identifies and quantifies the diamagnetic signatures of Aβ and τ tangles in AD, and the paramagnetic properties of iron. Studies on AD have focused on the paramagnetic properties of iron accumulation in the brain. However, recent studies highlight the importance of considering the diamagnetic properties of Aβ and τ aggregates, which can contribute to detectable changes in magnetic susceptibility [20, 21, 33]. Moreover, Studies using QSM have demonstrated its ability to visualize individual Aβ plaques ex vivo. The negative contrast observed in the susceptibility maps corresponding to plaque locations confirms their diamagnetic nature [21].

Distinguishing diamagnetic signals of Aβ and τ aggregates from other sources of susceptibility, such as myelin, requires advanced QSM techniques. Voxel‐vise susceptibility alteration separation methods allow the isolation of diamagnetic and paramagnetic contributions [12, 51, 52, 53]. This separation enables a more specific assessment of protein accumulation, independent of other susceptibility sources. Diamagnetic component susceptibility, obtained through source separation, can further quantify these changes, providing a measure of the Aβ and τ burden in the brain. However, there are challenges associated with utilizing diamagnetic signatures of Aβ and τ for AD diagnosis. The relatively small magnitude of the diamagnetic signal compared to paramagnetic iron can make detection difficult [19, 21]. High‐resolution imaging and sophisticated analytical techniques are required to ensure accurate identification and quantification [55]. Standardization of QSM acquisition and analysis protocols is crucial to ensure the reliability and reproducibility of findings across different studies [55].

Further supporting the value of QSM in AD research, studies have shown that QSM can detect alterations in white matter before significant atrophy is visible on conventional MRI [9, 25]. This early detection capability is crucial for timely intervention and can potentially slow disease progression. Additionally, the correlation of QSM measurements with clinical manifestations and Aβ deposition strengthens its potential as a valuable biomarker for AD [9, 34]. For example, studies have shown that paramagnetism in the gray matter nuclei, particularly in the globus pallidus and putamen, could serve as a marker of cognitive decline [32, 58]. Notably, one study reported high sensitivity and specificity (90% and 100%, respectively) of globus pallidus paramagnetism in AD diagnosis [60].

Recent advancements in QSM technology, particularly the introduction of paramagnetic‐diamagnetic signal separation techniques, have paved the way for more precise observation of AD protein aggregation and white matter demyelination. These innovations address a critical limitation of traditional QSM, where the overlapping presence of paramagnetic iron with diamagnetic Aβ and τ proteins complicates the interpretation of the susceptibility signals. Aβ and τ, which are diamagnetic, can be effectively visualized using QSM. Phantom experiments and analyses of AD brain specimens have validated that Aβ plaques and τ tangles possess diamagnetic properties, producing distinct contrasts in susceptibility maps [21]. Moreover, diamagnetic QSM signals have been shown to correlate closely with AD pathology. Research indicates that the magnitude of diamagnetic susceptibility in specific brain regions corresponds to the extent of Aβ and τ deposition, as well as the severity of cognitive decline in patients with AD [61]. This suggests that the diamagnetic QSM could serve as a valuable tool for tracking white matter damage. For instance, reduced diamagnetic signals in white matter, indicative of demyelination, have been observed in patients through techniques such as DECOMPOSE [51] and APART [52]. However, it is important to acknowledge that no advanced neuroimaging technique is capable of capturing all relevant information comprehensively, underscoring the need for further exploration through multiparametric and multimodal imaging studies [62].

The concentration of myelin in certain brain regions, such as cortical layers, is notably low [61]. However, the current QSM cannot differentiate between various metals and myelin contents within the brain. Additionally, QSM measurements can be influenced by potential variations in the diamagnetic myelin content. Another challenge lies in interpreting diamagnetic susceptibility maps, as they may contain contributions from non‐myelin sources [41] and iron‐containing species [63, 64] besides Aβ and τ. Future research efforts should prioritize the development of advanced QSM techniques or analytical methods capable of independently isolating and quantifying Aβ and τ signals. Potential approaches include exploring different magnetic field strengths or developing novel contrast agents that selectively enhance the diamagnetic signal of either Aβ or τ. Another direction is to combine QSM with other imaging modalities such as PET, which can provide specific information on Aβ and τ deposition, allowing for a more comprehensive and accurate assessment of AD pathology. Although diamagnetic QSM shows promising results in detecting Aβ and τ pathology, further large‐scale studies with optimized standardization of QSM acquisition and analysis [55] are needed to establish its sensitivity and specificity compared to gold‐standard methods such as PET.

## 3 Conclusions

Both paramagnetic and diamagnetic substances play crucial roles in AD pathology and progression. Although protein aggregation and white matter demyelination primarily manifest as changes in diamagnetic signatures, iron deposition contributes to the paramagnetic profile of the AD brain. Our primary review highlights the potential of QSM in advancing our understanding of AD pathology. By focusing on the diamagnetic properties of Aβ plaques and τ tangles, QSM offers a novel approach to visualizing and quantifying these key pathological features. The ability of QSM to act as a fully quantitative approach to differentiate between diamagnetic and paramagnetic substances provides a unique advantage in studying the complex interplay between Aβ, τ, and iron accumulation in the brain. Our findings suggest that the diamagnetic signatures of Aβ and τ detectable through QSM could serve as valuable biomarkers for AD diagnosis and progression monitoring. The development of advanced voxel‐wise and sub‐voxel QSM techniques enhances the sensitivity and specificity of detecting these protein aggregates, even in the presence of confounding paramagnetic substances such as iron. However, challenges remain in standardizing the QSM acquisition, signal processing, and analysis protocols to ensure consistent and reliable results across studies. Future research should focus on refining the QSM methodologies and exploring their clinical applications. The integration of QSM with other imaging modalities such as PET could provide a more comprehensive understanding of AD pathology and improve early diagnosis and intervention strategies. Ultimately, the insights gained from QSM studies have the potential to contribute significantly to the development of targeted therapies and improve AD management.

## Author Contributions

**Sadegh Ghaderi:** conceptualization, methodology/study design, software, data curation, writing – original draft preparation, visualization, investigation, validation, writing – reviewing, and editing. **Sana Mohammadi:** data curation, writing – original draft preparation, visualization, investigation, validation, writing – reviewing, and editing. **Farzad Fatehi:** supervision, validation, writing – reviewing, and editing.

## Ethics Statement

The authors have nothing to report.

## Conflicts of Interest

The authors declare no conflicts of interest.

## Data Availability

This article contains all the data produced or analyzed during this investigation. Further inquiries should be forwarded to the corresponding author.
